# Supplementary material for: Are carbon emissions trading and green financial instruments synergistic? -Comprehensive quantitative research based on content analysis
Source: PLoS One. 2024 Mar 7;19(3):e0298601. doi: 10.1371/journal.pone.0298601 (PMC10919601; doi:10.1371/journal.pone.0298601)
Supplement: S1 Data — (ZIP) [file pone.0298601.s001.zip › Data/地级市Z_F工作报告.zip!/║■─╧/│ñ╔│╩╨/└┤╫╘╣1⁄2╓┌║┼í╛┬φ┐╦╩2╛▌═°í┐_2012.docx]

各位代表：

　　现在，我代表市人民政府向大会报告工作，请予审议，并请各位政协委员和其他列席人员提出意见。

　　一、2011年工作回顾

　　2011年是形势复杂、困难较多的一年，也是攻坚克难、奋发有为的一年。在市委的坚强领导下，全市上下紧紧围绕“率先建成‘两型’城市和实现全面小康”的目标，科学应对，积极作为，圆满完成了市第十三届人民代表大会第四次会议确定的目标任务，实现了“十二五”的良好开局。

　　——经济发展再上新台阶。地区生产总值跨越5000亿元，达5600亿元（预计数，下同），增长14％以上；辖内财政收入迈过千亿大关，达1330亿元，增长28.55％，地方财政总收入668.11亿元，增长30.67％；完成固定资产投资3500亿元，增长20％以上；实现社会消费品零售总额2138亿元，增长18％。经济实现逆势增长。

　　——结构调整形成新格局。优势产业集群持续发展，工程机械实现产值1800亿元，三一重工跻身世界上市公司500强；食品烟草晋级千亿产业集群；战略性新兴产业异军突起，蓝思科技进出口额跃居全省第一；第三产业加快发展，对经济增长的贡献率同比提高4.8个百分点。产业格局正向多点支撑转变。

　　——现代农业取得新进展。实现粮食生产“八连增”,达到244.5万吨；花卉苗木、有机茶叶、优质水稻、特种养殖等特色产业继续发展；新增农业产业化国家重点龙头企业3家，农产品加工销售收入突破600亿元；专业合作组织蓬勃发展，休闲农业、观光农业快速发展；落户现代农业项目280个。现代农业成为农民增收、农村发展的重要动力。

　　——基础设施建设实现新突破。重大项目进展顺利，营盘路湘江隧道建成通车，过江交通压力明显缓解；黄花机场新航站楼投入运营；地铁1、2号线，福元路大桥，湘府路大桥，南湖路隧道建设顺利推进；湘江综合枢纽一期、沿江防洪道路工程完工；先导区“六纵八横”路网全面形成。重大基础设施建设为长远发展提供有力支撑。

　　——民生保障达到新水平。城乡居民人均可支配收入分别达26235元和12450元，分别增长15％和17％，居中部省会城市首位；着力解决上学难、看病贵的问题，基础教育全面发展，基本药物制度全面实施，居民基本养老、医疗保险制度城乡统筹全覆盖，两次提高城乡居民最低生活保障标准。城乡居民在发展中得到更多实惠，连续四年被评为中国最具幸福感城市。

　　——生态建设展现新风貌。“环境保护三年行动计划”全面完成。天马山、凤凰山和桃子湖“两山一湖”提质改造工程完工，洋湖湿地公园一期开园；城区61个排污口实现截污，湘江长沙段水质功能达标率100％；乡镇污水处理厂建设加快推进，长沙县实现集镇污水处理全覆盖；城市污水处理率达92.7％，城市建成区绿化覆盖率42.2％，空气质量优良率93.4％。城乡环境明显改观。

　　——成功创建全国文明城市。万众一心推进文明城市建设，经济社会协调发展，城乡面貌深刻变化，城市品质明显提高，人民生活不断改善，文明程度大幅提升，名列参评省会、副省级城市首位，成功夺得全国文明城市桂冠，实现了全市人民多年的夙愿。

　　一年来，我们突出抓了以下八个方面的工作：

　　一是突出重大项目建设。深入开展“两帮两促”专项服务，实施三“十”重大工程项目96个，完成投资402.7亿元。沪昆高铁、城际轨道建设顺利推进，长浏、大浏、浏醴、长韶娄等高速公路建设全面提速。梅溪湖国际新城成功整体招商，湖泊及景观工程基本完成。九龙仓、万达、世茂等城市综合体加快推进。晟通科技新生产线、介面光电等重大产业项目竣工投产，比亚迪一期、广汽菲亚特、广汽三菱、陕汽二期即将投产，住友轮胎、中电软件园等项目快速推进，成功盘活经阁铝材、曙光电子。天心文化产业园获批国家文化产业示范园，中南国家数字出版基地落户长沙。新引进戴尔信息、哈里斯等7家世界500强企业，瑞吉、万豪等6家国际品牌酒店进驻长沙。着力破解项目融资难题，市场直接融资305.4亿元，新增上市公司4家。实际到位外资26亿美元，引进市外境内资金850亿元，分别增长16.2％和27.1％。

　　二是突出转变发展方式。工业经济成为经济发展的主要支撑，工业总产值突破7000亿元；园区工业快速发展，高新区、经开区晋级千亿园区，生物医药产业基地电子信息与生物医药产业竞相发展；完成战略性新兴产业项目投资320亿元，高新技术产品增加值达980亿元，增长35％以上。加快创新型城市建设，新增国家重点实验室2家、国家级工程技术研究中心1家，成立“中国高校技术转移中心长沙联盟”；实施知识产权战略，专利申请数达1.2万件；第五届“科交会”签约193.57亿元，荣获全国促进城市发展科技奖。推进质量振兴和标准化战略，新增驰名商标10件，总量居中西部城市首位。大力发展现代服务业，一力、全洲、国药等重大物流项目竣工运营；实现服务外包业务总量300亿元、旅游总收入582.2亿元，分别增长26％和23.8％。非公有经济占地区生产总值60.1％。成功举办环湘江自行车赛、亚洲沙滩排球巡回赛、工程机械配套博览会、国际广告节等重大活动；开通长沙至新加坡国际航线，与加拿大维多利亚市结为友好城市，完成进出口总额75亿美元，增长23.2％。

　　三是突出宜居城市建设。注重用国际理念引领城市规划、设计和管理，实行规划修改前置审查，持续加强建筑方案审查和城市色彩管理。实施“沿江建设、跨江发展”战略，望城撤县改区，城区面积增加一倍，拓展了城市发展空间。黎托新城、新河三角洲、南湖等重点片区建设稳步推进。大力实施保障性安居工程，加快黄兴北路、大古道巷、九尾冲、长重等棚户区改造，完成棚户改造15887户，建成保障性安居房3.79万套。推进交通治堵，36条主次干道全线禁停，优化调整公交线路17条，填补公交空白路段32.5公里；开展高架桥、立交桥下综合整治，新增停车位8150个。实施黄花国际机场至市区沿线“一点两线”综合整治，周边环境显著改善；城市主干道两厢外立面整治基本完成，浏阳河西岸生态景观带芙蓉段建成开放，圭塘河生态景观区启动建设，313条城区道路路名牌焕然一新。加强宜居社区建设，完成老旧社区环境综合整治17个，7个城中村改造试点有效推进，提质改造支路街巷50条，建成景观街6条；新建社区公园30个，荣获全国人居环境范例奖。

　　四是突出新农村建设。坚持城乡统筹发展，实施农村电网扩容、公路通达、饮水安全、环境整治、校舍改造等“五大工程”。完成农村电网改造投资5.1亿元；新建、改造农村公路1500公里，改造危桥32座；开展全国小型农田水利建设示范县创建，完成小型水库除险加固35座，山塘清淤扩容1.5万口，解决31.3万人饮水安全问题，完成水土流失综合治理18.7平方公里。改造农村危房4250户、国有林场危旧房506户。林业综合改革纵深推进，森林覆盖率53.35％。莲花、雨敞坪等10个乡镇获评全国环境优美乡镇，宁乡县花明楼镇和长沙县印山村、开福区大明村、岳麓区山塘村被评为全国文明村（镇）。10个城乡一体化示范乡镇、31个试点示范村启动项目167个，试点示范取得阶段性突破。

　　五是突出“两型”社会建设。实施环境经济政策，积极探索以经济杠杆推动生态环保建设的新机制。强化能源审计和项目能评，试行“两型”社会城乡建设体系，宁乡经开区开展“两型”企业和“两型”园区创建试点。积极关闭和退出高能耗、高污染企业，完成长沙铬盐厂42万吨铬渣解毒处理工程，坪塘老工业基地污染企业全面退出。治理畜禽养殖污染40万平方米，启动大中型水库投肥养殖退出，天心区实现畜禽养殖业全面退出，宁乡县建立“退户入园、退村入场”养殖新模式。全面完成省政府下达的节能减排目标任务，成为全国首批节能减排财政政策综合示范城市。成立土地管理委员会，严格土地管理和供应，大力推进集约节约用地，加强耕地资源保护和闲置土地清理。推进地质灾害综合治理，大成桥沉陷安置小区一期竣工。规划实施第二个“环保三年行动计划”，被联合国授予“中国区环境规划示范城市”。

　　六是突出保障改善民生。坚持财政优先保障民生，全面完成为民办实事工作，全市民生支出达359.6亿元，增长24.5％。深入推进创业富民，新增城镇就业12.5万人、农村劳动力转移就业7万人，城镇登记失业率2.84％，零就业家庭连续多年实现动态清零。建立基础养老金正常调整机制，全市1.46万名老工伤人员纳入工伤保险统筹管理，“三无”人员和重症精神病人实现免费住院医疗，被征地农民参照城镇企业退休人员享受养老保险、医疗保险。发展慈善事业，慈善基金达1.3亿元，居中西部省会城市首位。深化医药卫生体制改革，全市连续十年无源发性药品安全质量事故。深入推进教育强市，着力保障校舍安全、校车安全，完成中小学校舍安全工程任务，望城区创造全国校车规范运营新模式；突出解决入学难、入托难，新建公办幼儿园37所，岳麓区成为全国城区公办幼儿园发展先进；职业教育快速发展，高等教育持续进步。创建国家公共文化服务体系示范区，完成示范性乡镇综合文化站、社区文化活动中心、农家书屋、广播电视户户通工程建设。推行城区中小学体育场地设施假日免费向社会开放，新建全民健身场所260个，获第7届城运会金牌6枚。落实“菜篮子”市长负责制，实行低保对象价格临时补贴、重度残疾人生活补贴，提高高龄老人生活补贴标准；加强价格监管，重要农副产品价格基本稳定。推进食品安全城市建设，实施放心猪肉、放心蔬菜、放心作坊、放心食堂工程，雨花区率先全省启动生猪产品质量安全溯源体系。

　　七是突出创新社会管理。开展“治安、治差、治堵、治难”专项行动，推进全国社会管理创新综合试点。完善“打、防、管、控”工作体系，社会治安形势总体平稳。开展流动人口均等化服务试点，人口自然增长率为4.28％。，被评为全国计划生育工作先进单位。用群众工作统揽信访工作，定期开展领导干部公开接访，化解一大批信访积案。完善应急管理体系，推进安全生产标准化，狠抓消防安全，创建全国综合减灾示范社区18个。在全市233个开放式社区中开展社区物业服务，开福区“社会管理社区化、社区管理社会化”管理模式初见成效。探索向公益类社会组织委托购买服务。圆满完成第六次全国人口普查和第八次村委会换届选举。落实民族政策，依法管理宗教事务。国防教育和动员工作成效明显，专职人民武装干部比武勇夺全省第一，荣获全国双拥模范城“五连冠”。

　　八是突出政府自身建设。自觉接受人大和政协监督，积极落实人大决议、决定，认真办理人大代表建议、批评、意见286件，办理政协提案540件。大力推行政府精细化管理，强化重大项目投资管理和审计，审查政府重大合同68件，核减项目投资60多亿元，审减资金27.3亿元。推进事业单位岗位设置管理工作。继续实施高端人才引进计划，引进国际高端人才101名、团队17个，其中国家“千人计划”专家15人。加大信息公开力度，加强在线审批和电子监察，开展全程无偿带领代办服务。市政府门户网站绩效评估居省会城市政府网站首位。开展重大行政决策听证，建立第三方参与立法后评估模式，加强规范性文件清理，探索行政司法互动机制。促进产权交易市场化，实现国资监管全覆盖。

　　与此同时，全面加强各项工作，国家安全、外事侨务、人防、对台、档案、机关事务管理、市志、供销合作、公积金管理、气象、地震、移民等工作取得新成绩。工会、共青团、妇联、科协、文联、侨联、社科联、残联、贸促会、红十字会、关工委、老龄委及各民主党派、工商联等为长沙发展做出新贡献。

　　各位代表！市第十三届人大一次会议确定，到2012年全市实现地区生产总值4000亿元、地方财政收入500亿元、固定资产投资2800亿元、社会消费品零售总额2000亿元、城乡居民人均可支配收入分别达到25000元和11500元。经过全市人民的不懈努力，这些主要指标已经提前完成，其他指标接近完成。这些成绩的取得，是省委、省政府和市委正确领导的结果，是市人大、市政协监督支持的结果，是全市人民团结奋斗的结果。在此，我代表市人民政府向全市各族人民，向全体人大代表、政协委员，向各民主党派、工商联、人民团体和各界人士，向驻长部队、武警官兵和政法干警，向所有关心支持长沙发展的朋友们，表示诚挚的感谢和崇高的敬意！

　　回顾过去，我们深切地体会到：必须科学发展。始终把发展作为第一要务，以加快发展解决前进中的矛盾和问题。坚持民主决策，科学谋划，走理性务实的发展道路，重视品质，严格管理，不急功近利，严禁政绩工程、形象工程，既为当前发展打基础，又为长远发展增后劲，实现率先发展、科学发展、和谐发展。必须民生为先。始终把改善民生作为经济发展的主要目的，高度重视群众切身感受，将群众冷暖记在心头，看问题、想办法、定政策首先考虑群众需求，从群众关心的事情做起，从群众不满意的事情改起，切实解决好就业、住房、医疗、入学等实际困难，让群众生活得更加美好、更加幸福。必须拓展视野。一个现代化的城市，首先是一个开放的城市，必须具有开放的市场、开放的文化。坚持以国内领先、国际先进水平为坐标，以国际视野指导建设发展，用现代理念推动政府工作，克服地域观念，建立公开、公平、开放的市场，鼓励一切有实力、有技术的队伍参与竞争，引进、吸收、利用国内外先进技术、管理经验、文化成果，在竞争中促进本地产业的发展和壮大，建设充满活力、开放包容的长沙。必须敢于担当。牢固树立执政为民的理念，珍惜人民赋予的权利和服务的机会，全心全意为人民谋福祉，不谋任何私利，敢于坚持原则，勇于担当责任，不回避矛盾，不畏惧困难，为了长沙的发展，为了公共利益，不怕得罪人，不计个人得失，义无反顾。必须精细管理。细节决定成败。高效廉洁的政府必须靠法律、制度和程序来保障，富有内涵的现代化城市必须靠规划建设的每个细节来实现。坚持把制度建设作为精细化管理的基础工作，以制度管人管事管权，从大处着眼、小处入手，把大事干对、小事干好，在城市建设、政府投资、公共财政、资源管理各个领域全方位推行精细化管理，降低行政成本，提高行政效率。实践证明，这些措施是行之有效的，必须倍加珍惜，在今后的工作中一以贯之地坚持和发扬。

　　在总结成绩的同时，我们也清醒地看到前进中存在的困难和问题：经济增长的资源环境约束趋紧，转变发展方式的任务十分艰巨；农村公共服务水平总体较低，促进农民持续增收的压力仍然较大；城市管理水平有待进一步提高；物价上涨给弱势群体和困难群众生活造成影响，就业、出行、教育、医疗、住房等民生问题有待进一步解决；个别领域和地方安全生产形势依然严峻，社会治安防控体系有待进一步完善；行政效能与群众要求还有差距等。这些问题，我们将采取有效措施，切实加以解决。

　　二、2012年面临的形势和任务

　　2012年是实施“十二五”规划的关键一年。中央经济工作会议对今年国际国内形势进行了全面分析，世界经济形势总体上仍将十分严峻复杂，我国经济虽然继续朝着宏观调控预期方向发展，但经济增长下行压力和物价上涨压力并存。因此，今年将是既复杂又困难的一年。我们的发展同样面临着巨大压力：一是经济下行的压力。受国际金融危机和国内消费需求不足的影响，去年下半年以来，全市经济增长呈现逐月下降的态势。由于国家继续实施稳健的货币政策，坚持房地产调控，投资增长特别是房地产投资面临巨大压力，加之外需疲软，消费拉动乏力，经济增长存在下行的风险。二是结构调整的压力。近年来全市产业发展虽然取得了长足进步，但经济增长主要依赖投资拉动的状况短期内仍将难以改变；传统产业技术改造投入不足，战略性新兴产业规模不大，经济增长的科技支撑力不强，节能减排形势依然严峻；生产成本不断上涨，使实体经济受到冲击，形成了结构调整的倒逼压力。三是要素保障的压力。由于银行信贷受控，政府融资平台功能受限，加上市财政进入偿债高峰期，保民生、保运转、促发展的压力增大；长沙能源消费集中于煤炭和电力，由于煤电价格倒挂，调煤保电更加困难；征地拆迁直接制约项目推进，土地供应难以满足项目建设需要。资金、能源、土地成为亟需破解的发展瓶颈。四是民生改善的压力。经济增长放缓增加了就业压力，物价上涨使困难群体生活受到影响，群众对民生保障有更高的期待。同时，利益诉求多元化，利益格局复杂化，一些深层次矛盾相继浮现，加强和改善民生、维护和谐稳定的任务依然繁重。

　　各位代表！虽然我们面临巨大的挑战，但挑战中蕴含着机遇，我们更要看到发展的有利条件。一是积累了应对危机的宝贵经验。国际金融危机以来，市委、市政府及时谋划、科学应对，持续开展“两帮两促”活动，抢抓机遇干成了一批多年想干而没有干成的大事，实现了又好又快发展。成功的实践为我们应对新的危机提供了有益借鉴。二是奠定了加快发展的坚实基础。经过多年的持续快速发展，长沙经济总量不断增长，综合实力显著增强，城市形象明显提升，正在成为具有强大吸引力、辐射力和带动力的区域中心城市。特别是近年来实施了一批拉动作用大、支撑能力强、群众受惠多的重大项目，这些项目有的正在加快建设，有的开始发挥效益，增强了发展后劲。三是形成了区域竞争的独特优势。长沙是国家实施中部崛起战略和“两型”社会建设综合配套改革试点核心城市，也是国家首批节能减排财政政策综合改革示范城市和节能与新能源汽车示范推广试点城市，政策优势更加明显；随着黄花机场新航站楼投入使用和武广高铁开通，沪昆高铁和城际轨道开工建设，高速公路网络不断完善，区位优势更加突出。

　　同时，国家适时适度实施微调预调、加大水利建设投入、加强保障性住房建设、推动文化大发展大繁荣，为我们战胜困难提供了机遇。特别是省委、省政府高度重视和大力支持省会建设，为长沙发展增添了巨大动力；全市人民热切期盼又好又快、率先发展，形成了加快发展的强大合力。我们完全有理由、有信心实现经济社会平稳快速发展。

　　各位代表！省第十次党代会、市第十二次党代会描绘了未来五年发展的宏伟蓝图，我们要大干新五年，实现大发展，城乡大变样，再上新台阶。今年是贯彻落实省、市党代会精神的第一年，政府工作的总体要求是：以邓小平理论、“三个代表”重要思想为指导，深入贯彻科学发展观，全面落实中央、省、市委经济工作会议精神，按照“城市国际化、产业高端化、城乡一体化、发展‘两型’化、管理法治化，率先建成‘两型’城市和实现全面小康”的要求，坚持稳中求进、创新突破，又好又快、率先发展，紧紧围绕“稳增长、控物价、调结构、惠民生、抓改革、促和谐”的目标，加快转变发展方式，大力保障和改善民生，全面推进创业之都、宜居城市、幸福家园建设。

　　今年经济社会发展主要预期目标是：地区生产总值增长13％；财政总收入增长15％；固定资产投资增长20％；社会消费品零售总额增长18％；单位地区生产总值综合能耗下降3.5％，化学需氧量、二氧化硫、氨氮、氮氧化物等减排指标完成省下达任务；城乡居民人均可支配收入分别增长12％和14％；城镇登记失业率在4％以内；人口自然增长率在6.9％。以内。

　　实现上述目标，工作中必须着重把握以下几点：

　　一是坚持在稳定增长中实现率先发展。稳是前提，进是主题。科学应对危机，就是要保持经济稳定增长，防止经济下滑，为保持社会大局和谐稳定提供经济保障。从长沙来看，在日趋激烈的区域竞争中，前有标兵、后有追兵，这就决定了在相当长一段时间内必须把发展作为第一要务，保持经济持续快速发展，实现在全省率先、中部领先、全国争先的发展目标。

　　二是坚持在加快发展中调整经济结构。没有一定的增长速度和总量扩张，结构调整就会失去基本动力和支持空间。不能静止地调整经济结构，必须加快发展速度，依靠增量扩张消化淘汰落后产能带来的就业、税收减少，增强企业推进技术改造和自主创新、发展战略性新兴产业的动力，促进经济增长由依靠资源消耗向依靠科技进步转变，实现经济总量做大、均量提高、质量提升。

　　三是坚持在结构调整中突出发展实体经济。实体经济是推动发展的根本力量。目前，实体经济受外需疲软和内需不足的冲击，发展面临诸多难题。调整经济结构就是要重视以工业经济为主体的实体经济发展，加大对实体经济发展的政策支持力度，特别是支持中小企业发展，引导社会资本更多地投向实体经济，优化经济发展环境，促进实体经济平稳健康发展。

　　四是坚持在城乡统筹中加强农业基础地位。“三农”问题始终是发展和建设的全局性、根本性问题。坚持把解决“三农”问题作为全部工作的重中之重，按照城乡统筹发展的要求，加大农村基础设施建设投入，引导工业反哺农业，积极实施科技兴农，大力发展现代农业，促进农村发展、农业增效、农民增收。

　　五是坚持在住房保障中促进房地产业发展。房地产业是民生产业，对经济拉动的链条长，对经济增长的贡献大，房地产业大起大落不利于经济发展和民生改善。要认真落实中央宏观调控政策，加强监控管理，防止投机推高房价、形成泡沫。同时，加快保障性住房建设，探索农民拆迁安置消化存量住房，加快实现“住有所居”的目标。

　　三、2012年工作重点

　　面对复杂多变的发展形势，要保持经济社会发展的良好势头，必须着力抓好以下九个方面的工作：

　　（一）推进投资稳定增长。突出重大项目建设。继续实施重大项目带动战略，以重大项目为载体优化投资结构，确保有效投资增长。集中力量抓好沪昆高铁、城际轨道等中央、省在长项目建设，加快建设地铁、湘江综合枢纽、比亚迪、陕汽配套园等在建、续建重大项目。力争全年实施重点项目566个，完成投资1494亿元。创新融资方法。坚持政策性融资与市场融资相结合，拓展融资渠道。加强衔接申报，最大限度争取国家项目支持。鼓励和引导金融机构落户长沙，有序发展小额贷款公司和村镇银行；发挥上市公司的融资作用，支持优质企业上市融资。充分发挥先导控股、城投集团投融资功能。推进科技金融深度融合，设立创新创业引导基金，引导发展创业投资机构。落实扶持政策，激活民间投资。优化土地供应。认真研究土地市场的新需求、拆迁政策的新变化和被征拆群众的新诉求，坚持依法征收、阳光拆迁；坚持节约集约用地，积极推广节地模式，合理有序供应土地，依法处置闲置用地，多措并举破解用地瓶颈。同时，采取有效措施，确保煤电油气等要素供应。

　　（二）推进产业协调发展。加快新型工业化步伐。坚持把新型工业化作为第一推动力，加快发展工程机械、汽车及零部件等优势产业，积极培育新材料、电子信息等战略性新兴产业，集中打造千亿产业集群。着力提升“三区九园”承载功能，支持高新区创建国家自主创新示范区，推动经开区、浏阳工业新城、宁乡金洲新区等园区提质扩容，加快岳麓科技产业园规划建设。落实国家支持中小企业发展政策，实施中小企业知识产权战略推进工程。加强产业技术创新战略联盟建设，推进企业科技创新，提升企业竞争力。大力发展现代服务业。重点发展生产性服务业，提升发展传统服务业，积极拓展新型服务领域，不断培育新的服务业态。加强配套服务，促进现代物流、电子商务、服务外包、物联网健康发展。培育金融商务产业集群，推进区域性金融中心建设，加快芙蓉中央商务区、空港新城、金融生态区等现代服务业集聚区发展。推进河西商业配套建设，推动马王堆、红星市场外迁。着力改善城乡消费环境，培育新的消费热点，扩大消费需求。扶持发展现代农业。稳定粮食生产，发展蔬菜产业，加快重点蔬菜基地建设。支持都市农业、休闲农业发展，推进雨敞坪、莲花、柏加和白箬休闲农业示范片建设。支持油茶、花卉苗木和林下经济产业发展。加强基层农业技术推广体系建设，培育壮大农村经济合作组织。加快望城国家农科园、宁乡农产品加工创业园和浏阳农业园建设。

　　（三）推进宜居城市建设。坚持高起点规划设计。完成城市总体规划修改报批工作，按照建设现代化、国际化城市的要求，优化城市色彩、城市轮廓线、标志性建筑设计，增强规划的前瞻性和约束性。完善望城区功能定位、空间规划和产业布局，加快大河西建设，形成“一江两岸”互动发展的城市空间格局。坚持高密度组团式发展，加快高铁、地铁、城际轨道沿线开发。推进滨水新区、隆平新城、滨江新城、南湖片区、金霞片区等重点片区特别是黎托新城开发建设。加速推进黄兴北路、长重、九尾冲等棚户区改造。提升城市承载功能。启动荷花路浏阳河隧道、红旗路、东二环线改造工程；开工建设地铁2号线西延线和3、4号线一期工程；加快南湖路湘江隧道、人民东路东延线、潇湘大道北延线建设；完成银盆岭大桥加固改造，实现湘江综合枢纽蓄水通航，湘府路大桥、福元路大桥建成通车。实施先导区新三年行动计划，加快梅溪湖国际服务区、洋湖总部经济区、大河西综合交通枢纽等重大项目建设，全面提升先导区示范功能。实施公交优先战略，启动公交企业改革重组，优化公交站点布局，加快公交站场及设施建设。巩固文明城市创建成果。全面启动创建国家卫生城市，健全文明城市长效管理机制。深化城管体制改革，建立“大城管”联席会议制度，基本实现数字城管。积极探索城市管理市场化服务模式，创建200个社区物业服务工作示范社区。突出城乡结合部环境综合整治，打造城市出入口特色风貌；加快“城中村”改造步伐，全面提质老旧社区、老旧厂区环境，推进社区公园建设。努力建设更高水平的文明城市。

　　（四）推进城乡统筹发展。加速城乡基础设施对接。加快实现城乡一体化，促进城乡共同繁荣。推进望城区基础设施与主城区无缝对接，实现全面融城；加快宁乡县与主城区、金洲新区与高新区对接步伐；推动长沙县临主城区边际规划建设一体化；加快浏阳市连接主城区的快速通道建设，促进浏阳工业新城与长沙经开区对接，推动浏阳融入省会半小时经济圈。继续实施五大工程。加强基本农田水利建设，推进大中型水库库区、河道流域治理，完成28座中小型病险水库除险、5万亩山塘清淤增蓄，完成城区新、改建供水工程4处，解决30万农村人口饮水安全问题；继续开展农村电网改造升级工程；加强干线路网和农村公路建设与维护，硬化通村公路1500公里；建设16家乡镇污水处理厂，建成沼气池6万立方米；切实改善农村学校食宿条件。推进小城镇集约发展。加强乡镇、村规划建设管理，编修城镇总体规划和村庄规划。实行小城镇规划、建设、国土、环保、城管“五位一体”管理新模式，创建20个示范小城镇。继续重点支持城乡一体化示范镇、新农村示范村建设，更加重视边远乡镇和村级集体经济发展，加大对口支援力度，增强发展造血功能。完善农村公共服务。加大对农村的财政转移支付，完善农村医疗救助制度，扩大农村低保、医保和养老保险覆盖面，提高农村“五保户”保障水平。完善农村基本卫生服务，加强偏远贫困乡镇卫生院和村卫生室建设。健全被征地农民社会保险、再就业、保障性住房建设机制，逐步实现城乡基本公共服务均等化。

　　（五）推进文化繁荣发展。建设国际文化名城。坚持用国际视野提升城市文化形象，保护和发掘城市历史文脉，彰显湖湘文化特质。实施重大文化建设工程，重点推进市图书馆、博物馆和音乐厅建设，加快长沙近现代史博物馆、市青少年宫改造和建设，打造具有长沙地域风情的城市标识。加大非物质文化遗产保护力度，加强古镇、古村落、古民居的保护，推进炭河里、汉长沙王、铜官窑考古遗址公园建设。优化公共文化服务。推进国家公共文化服务体系示范区建设，提质改造乡镇（街道）综合文化站，加强社区公共电子阅览室建设。大力开展送戏、送电影、送图书、送文艺节目等文化下乡、进社区活动。积极引导和规范民间文化组织、行业协会发展，重视支持基层文化队伍建设。做大做强文化产业。坚持文化产业融合发展、创新发展，培育壮大影视传媒、动漫游戏、新闻出版、文博会展、演艺娱乐等特色文化产业，打造一批有实力、有竞争力、有影响力的文化企业，推进天心文化产业园、岳麓大学城等文化创意集聚区发展。深化文化体制改革，加快市直文化单位改制，引导规范文化、体育市场发展。

　　（六）推进综合配套改革。开展节能减排综合示范。实施重点领域节能减排项目工程，大力推进技术创新。严格实施项目能评制度，先导区范围内落后产能、工艺和设备淘汰率达到100％，城区范围内污染严重的企业100％关闭退出或搬迁改造。推进节能文化进家庭、入学校，深入开展全民节能行动。实施新一轮“环保三年行动计划”。加强湘江长沙段和浏阳河、捞刀河、靳江河、沩水河环境综合整治，新建和扩建暮云、长善垸、花桥等污水处理厂，实现城区排污口全截污，确保湘江综合枢纽蓄水通航后饮用水源地水质安全。大力实施林业生态工程，继续开展岳麓山景区综合整治，启动坪塘工业基地生态修复和桃花岭景区、大王山公园建设，加快西湖文化园、洋湖湿地公园二期建设，建成圭塘河生态景观区。加强噪声、渣土扬尘和机动车尾气治理，逐步淘汰城区“黄标车”，空气质量优良率保持在92％以上。城市禁养区畜禽养殖业全面退出，农村养殖逐步推行“退户入场、退村入园”；推行农村垃圾集中处理，巩固农村环境综合整治成果。完成黑麋峰垃圾填埋场提质改造，建成餐厨垃圾、污泥处理项目和危险废弃物处置中心。推进重点领域改革。加快建立生态补偿机制，全面推行主要污染物排污权使用和交易，建立健全“两型”社会和生态文明建设综合评价体系。开展农村土地、人口户籍、农村金融等重点改革。建立耕地保护补偿机制，规范农村土地流转。

　　（七）推进对外开放合作。加大招商引资力度。坚持引资与引智、引项目与引专利相结合，创新招商方式，提高招商引资质量。始终瞄准世界500强、跨国公司、大型央企，引进具有带动性、基础性、全局性的投资项目和领军人才与团队。积极扩大国有资本对外合作，抓好国企国资重点项目与央企对接。精心筹办重要节会，全面加强区域合作。加快外向型经济发展。加强外贸出口平台建设，以金霞陆路口岸作业区建设为重点，加快长沙综合保税区申报和建设，打造外贸转型升级示范基地和出口创新基地。构建灵活高效的“大通关”机制，帮助解决重点企业、重大项目的通关问题。促进现代旅游业发展。立足资源优势和区域特色，科学规划旅游产业功能区布局，把旅游业培育成战略性支柱产业。坚持政府主导、精品建设，以湘江综合枢纽蓄水通航为契机，加快推进湘江旅游带的开发建设。支持灰汤温泉创建国家旅游度假区、大围山创建国家生态旅游示范区，打造一批旅游综合体、风情街区和特色村镇，推出一批精品旅游线路，将长沙建设成为世界旅游目的地。

　　（八）推进民生事业发展。完善社会保障体系。坚持以创业带动就业，争创国家创业型城市，着力为每一个有劳动能力的人创造就业机会，保持城镇零就业家庭动态清零。探索建立基本养老金正常调整机制，实现各险种市级统筹，社保参保率达95％以上。加快社会救助体系建设，关爱特困群众、残疾人群体。实施“惠老工程”，创新养老服务模式，改扩建乡镇敬老院20所，创建省级农村综合性老年福利中心示范工程6个。完善住房保障体系，新建（筹集）公共租赁房1.4万套，新建廉租房2258套。狠抓“菜篮子”工程，开展农超对接，千方百计稳定蔬菜价格。加快建设教育强市。合理配置公共教育资源，推进义务教育标准化学校建设。加强城市开发配套基础教育建设，着力解决进城务工子女就学和城区部分学校“大班额”问题。新建、改扩建公办幼儿园35所，引导和支持民办幼儿园提供幼儿普惠教育服务。推动市属高校与知名院校合作共建。加大示范性职业院校创建力度，大力发展面向市场、对接产业的职业教育。规范社会力量办学，整顿教育培训市场。推进健康长寿之城建设。加强公共卫生服务体系建设，探索公立医院改革，深化基层医疗卫生机构综合改革，新建一批社区卫生服务中心。落实国家基本药物制度，推行基层医疗机构药品网上集中采购、医疗机构双向转诊制度。加大重大传染疾病预防控制。深入推进食品安全城市建设，强化企业主体责任和部门职能职责，整合食品安全检测力量，加大联合执法力度，确保群众吃得安全、放心。广泛开展健康促进行动，提高全民健康水平。

　　（九）推进社会管理创新。实施社会管理法治化。加强社会主义法治理念教育，弘扬社会主义法治精神。建立社区群众工作站，加强社区综合管理中心和综合执法队伍建设。完善多元化的矛盾纠纷解决机制，畅通群众诉求渠道，加大信访历史遗留问题的解决力度，维护群众根本利益，依法维护信访秩序。加强司法救助和法律援助，为群众提供优质、高效、规范的法律服务。大力建设平安长沙。加快“天网工程”和应急联动指挥中心建设，积极预防和依法打击各种违法犯罪行为，提升社会治安防控能力，提高市民的安全感。严格落实安全生产责任制，突出加强消防安全，防止各类重特大事故发生。完善公共突发事件监测预警、应急处置机制，提高防灾减灾意识，保障人民群众生命财产安全。努力构建和谐长沙。开展“一推行四公开”活动，推行干部联点驻村（社区）、公开联系方式、公开岗位职责、公开监督机制、公开考核办法。加强基层社会管理服务体系和社会工作者队伍建设，全面完成社区换届选举，增强社区自治和服务功能。突出抓好流动人口、特殊人群、信息网络的服务管理，充分发挥非公有制经济组织和社会组织的积极作用。加强统计工作，推进统计改革。开展民生档案服务创新试点。落实企业工资集体协商制度，确保劳资和谐。加强诚信体系建设，倡导良好的社会道德风尚。做好人口与计划生育工作，保护妇女、儿童、老年人、残疾人合法权益。加强国防教育、人民武装、人民防空工作，推进国防后备力量体系建设。落实民族宗教政策，发挥宗教界人士积极作用，促进民族团结进步。促进国家安全、外事侨务、机关事务管理、地方志等工作全面进步。

　　四、切实加强政府作风建设

　　完成繁重的工作任务，实现经济社会又好又快发展，必须加强政府作风建设。

　　（一）牢固树立公仆意识。每一个政府工作人员都是人民的公仆，必须牢固树立公仆意识，始终牢记全心全意为人民服务的宗旨，始终坚持让人民满意的行为准则，时刻摆正自己与群众的位置。在思想上尊重群众，感情上贴近群众，行动上深入群众，主动为群众服务、为企业服务、为基层服务，用实干赢得群众的信任，用实绩回报人民的支持。

　　（二）始终关心群众利益。服务群众是政府工作的永恒主题。要始终把群众放在心坎上，永远与群众在一起，真正把群众当主人、当亲人、当老师。经常问政于民、问需于民、问计于民，想问题、做决策、安排资金，必须首先考虑群众受益，确保有限的财力用在群众最需要的地方。时常换位思考，设身处地理解群众的困难，了解群众的需求，帮助群众解决入学难、看病难、出行难，让群众得到更多的实惠。坚持一切从群众利益出发，做群众利益的代言人和保护者，坚决反对漠视群众利益、遇事推诿、甚至拿公共资源做人情的“老好人”作风。我们决不做“高高在上”的政府，要做实实在在为群众谋福祉的政府。

　　（三）更加注重基层建设。基层是联系群众的桥梁，是做好政府工作的重要依托。基层干部处在矛盾的第一线、问题的最前沿，条件艰苦、任务繁重、压力很大。要真正重视、真情关怀、真心爱护、热情支持。要实行重心下移，继续简政放权，积极为基层减负。要特别关心和支持边远地区基层建设，着力改善基层待遇和工作条件，真正让基层干部辛苦工作有甜头、服务基层有盼头、扎根基层有奔头。

　　（四）热忱支持企业发展。企业是创造财富的实体，没有企业的发展就没有解决民生的条件。越是经济发展困难的时候，越要关心支持企业发展，要更多地理解企业家创业的艰辛、成长的艰难，不断改进对企业的服务，着力帮助企业破解融资、供地、用电、招工等困难，为企业创新创业、加快发展营造更加宽松的政策和发展环境。只要重视企业、关心企业，企业就会更加主动地承担更多的社会责任，为长沙发展做出更大贡献。

　　（五）切实提高专业素质。复杂多变的发展环境和日益精细的社会分工，对政府工作人员的综合素质特别是专业素质提出了更高的要求。长沙要实现更高水平、更快速度的发展，政府工作部门都必须提高工作水平，所有工作人员都必须具有专业素养。继续加大人才工作力度，实施重大人才工程，开展专业能力和素质培训，努力锤炼一支素质高、能干事、敢担当、作风硬的干部队伍。政府工作人员要主动克服思想上的惰性、工作中的惯性，少开会、少应酬，多调查研究、多学习思考、多解决问题，真正做到干一行、爱一行，成为所在行业和领域的专家。

　　（六）着力推进依法行政。依法行政是建设法治政府的核心，是政府公信力的生命线，是政府工作的基本准则。加大行政立法力度，深入实施《湖南省行政程序规定》，规范行政审批程序。强化行政监督，自觉接受人大和政协监督，优质高效办理人大代表议案、建议和政协提案，认真听取和采纳各民主党派、工商联、人民团体和社会各界意见，主动接受新闻媒体和社会公众监督，严肃查处利用职权或公共资源谋取私利的腐败行为，建设诚信、廉洁的政府。任何时候都要做到决策有据、行政依法，确保政府的每一项决策都体现民主的力量，每一个行政行为都闪耀法制的光辉。

　　各位代表，今年是本届政府履职的最后一年。为人民服务只有起点没有终点，我们将一如既往地坚持党的利益至上，人民利益至上，敢于负责，勇于担当，在市委的坚强领导下，紧紧团结和依靠全市人民，迎难而上，开拓进取，创造更加优异的成绩迎接党的十八大胜利召开！
